# Identifying the risk of obstructive sleep apnea in metabolic syndrome patients: Diagnostic accuracy of the Berlin Questionnaire

**DOI:** 10.1371/journal.pone.0217058

**Published:** 2019-05-21

**Authors:** Felipe X. Cepeda, Leslie Virmondes, Sara Rodrigues, Akothirene C. B. Dutra-Marques, Edgar Toschi-Dias, Fernanda C. Ferreira-Camargo, Maria Fernanda Hussid, Maria Urbana PB Rondon, Maria Janieire N. N. Alves, Ivani C. Trombetta

**Affiliations:** 1 Heart Institute (InCor) do Hospital das Clínicas da Faculdade de Medicina da Universidade de São Paulo, São Paulo, Brazil; 2 Universidade Nove de Julho (UNINOVE), São Paulo, Brazil; 3 Universidade Ibirapuera, São Paulo, Brazil; 4 School of Physical Education and Sports, University of São Paulo, São Paulo, Brazil; Medical University of Vienna, AUSTRIA

## Abstract

**Background:**

Obstructive sleep apnea (OSA) is a risk factor frequently present in patients with metabolic syndrome (MetS). Additionally, moderate and severe OSA are highly prevalent in patients with cardiac disease, as they increase the riskfor cardiovascular events by 80%. The gold standard diagnostic method for OSA is overnight polysomnography (PSG), which remains unaffordable for the overall population. The aim of the present study was to evaluate whether the Berlin Questionnaire (BQ) is anuseful tool for assessing the risk of OSA in patients with MetS.

**Methods:**

97 patients, previously untreated and recently diagnosed with MetS (National Cholesterol Education Program, Adult Treatment Panel III, ATP-III) underwent a PSG. OSA was characterized by the apnea-hypopnea index (AHI). BQ was administered before PSG and we evaluated sensitivity, specificity, positive and negative predictive values, and accuracy.

**Results:**

Of the 97 patients with MetS, 81 patients had OSA, with 47 (48.5%) presenting moderate and severe OSA. For all MetS with OSA (AHI≥5 events/hour), the BQ showed good sensitivity (0.65, 95% CI 0.54 to 0.76) and fair specificity (0.38, 95% CI 0.15–0.65) with a positive predictive value of 0.84, a negative predictive value of 0.18 and an 84% accuracy. Similarly, for moderate-to-severe OSA (AHI≥15 events/hour) we found good sensitivity (0.73, 95% CI 0.58–0.85) and fair specificity (0.40, 95% CI 0.27–0.55). Interestingly, for severe OSA (AHI≥30 events/hour), there was a very good sensitivity (0.91, 95% CI 0.72–0.99) and moderate specificity (0.42, 95% CI 0.31–0.54).

**Conclusion:**

The BQ is a valid tool for screening the risk of OSA in MetS patients in general, and it is particularly useful in predicting severe OSA.

## Introduction

Metabolic syndrome (MetS) has been shown to increase the risk for cardiovascular disease and the fast-increasing worldwide prevalence of this condition is indeed worrisome [1]. MetS is characterized by visceral obesity, changes in blood pressure, glucose, triglycerides and HDL-c. Furthermore, MetS is present across all age groups, with highest prevalence in the elderly [2]. The association between MetS and obstructive sleep apnea (OSA) has been consistently found in the literature [3–5].

OSA is defined by a complete (apnea) or a partial (hypopnea) obstruction of the upper airway during sleep [6]. This anatomical change causes classic symptoms such as snoring, breathing pauses, and daytime sleepiness, which significantly impairs quality of life [7]. Sleep disordered breathing is closely related to obesity [8] and hypertension [9], two MetS risk factors. Indeed, MetS and OSA share some pathophysiologic conditions related to increased cardiovascular risk.

The association between MetS and OSA has been studied for over twenty years and it is also known as the "Z Syndrome" [10]. Such association can potentiate the likelihood of cardiovascular events due to the overlap of risk factors such as obesity [10], hypertension [9], diabetes mellitus 2 [11].

The gold standard to the diagnosis of OSA is nocturnal polysomnography (PSG), defined through the apnea-hypopnea index (AHI) [6]. However, patients need to stay overnight at a proper PSG laboratory, which is time-consuming and costly.

Albeit other screening tools as STOP-Bang questionnaire (SBQ), and STOP questionnaire (STOP) are frequently used for identifying OSA, the Berlin Questionnaire (BQ) has been the most used screening tool for this condition, and it has the highest number of validation studies [12]. BQ consists of 11 questions subdivided into three categories, assessing snoring, daytime tiredness and high blood pressure and/or body mass index (BMI), aiming at identifying some key OSA symptoms [13]. However, BQ accuracy varies with the population studied [14–18]. In this context, the accuracy of BQ in detecting the presence of OSA in patients with MetS remains to be clarified.

Another frequent used questionnaire for screening sleep-disordered breathing is the Epworth Sleepiness Scale (ESS). The eight question ESS is used to evaluate sleepiness during daily activities and not OSA *per se*, and a final score between 11 up to 24 points [19] means that a positive result was found, but this could be due to reasons unrelated to OSA. Thus, further studies are needed to establish the usefulness of this tool in different populations.

We attempted to determine the accuracy of the BQ in identifying the risk of OSA based on overnight PSG in patients with MetS. Our hypothesis was that the BQ is a user-friendly screening tool for OSA in patients with MetS.

## Materials and methods

### Study Population

This is a cross-sectional study. Between 2010 and 2016 we consecutively recruited 97 male and female patients, aged between 30–65 years, from the Outpatient Unit of the Heart Institute (InCor), University of São Paulo Medical School. During the recruitment process, all the patients were included if they met the inclusion criterion, i.e., a recent diagnosis of MetS, based on the National Cholesterol Education Program, Adult Treatment Panel III (ATP- III) [20]. MetS was characterized when the subjects met at least three of these five diagnostic criteria: (1) high-density lipoprotein (HDL) cholesterol <40 mg/dL (<1.03 mmol/L) in men and <50 mg/dL (<1.29 mmol/L) in women; (2) fasting glucose leve1 ≥l00 mg/dL (≥5.6 mmol/L); (3) fasting triglyceride level ≥150 mg/dL (>1.69 mmol/L); (4) waist circumference ≥102 cm in men and ≥88 cm in women; and (5) systolic and diastolic blood pressure (BP) ≥130 and/or 85 mmHg. We excluded from the sample patients who were taking medications, with a history of excessive alcohol consumption, smokers, and those who had a history of either cardiovascular or pulmonary disease. Approximately 65% of the studied patients had also participated in previous studies [5, 21].

#### Ethics and trial registration

The study was approved by the Scientific Commission of the InCor, and by the Ethics Research Committee of the University of São Paulo Medical School (#1222/05), and each subject signed a written consent form. This protocol followed the CONSORT Statement.

#### Experimental design

All evaluations were performed within 2 weeks, with interval of 2 days between 1st-2nd clinic visits and approximately 1 week between 2nd-3rd clinic visits. During the first clinic visit, all subjects underwent 3 standard blood pressure measurements, and assessment of body weight, height, body mass index (BMI) and waist circumference. They were then invited to participate in the study, and after providing a written informed consent, they underwent the evaluations to confirm MetS diagnosis.

In the second visit, venous blood was collected after 12 hours of overnight fasting to measure total serum cholesterol, triglycerides, HDL cholesterol (enzymatic method) and plasma glucose (standard glucose oxidase method).

In the third visit, the patients answered the BQ and ESS questionnaires and subsequently underwent PSG for a more accurate diagnosis of OSA at the same hospital.

### Procedures and measures

#### Overnight polysomnography

We evaluated the OSA by a full overnight PSG (Embla digital system, 17 channels, EMBLA, Flaga hf. Medical Devices, Reykjavik, Iceland) as previously described [21]. PSG started at around 10:00 PM and finished at around 6:00 AM. AHI was calculated as the total number of respiratory events (apneas plus hypopneas) divided by the total sleep time and expressed by events per hour (events/h). AHI cutoff for OSA was based on the Task Force of the American Academy of Sleep Medicine [22]. The severity of OSA was classified as such: non-OSA for AHI < 5 events/h, mild OSA for AHI from 5 to 14.9 events/h, moderate OSA for AHI from 15 to 29.9 events/h, and severe OSA for AHI ≥ 30 events/h [6]. Apnea was defined as a 90% decrease of airflow for at least 10 sec, while hypopnea was defined by > 50% decline in airflow in respiratory signals for at least 10 sec accompanied by 3% oxygen desaturation [22].

#### Berlin questionnaire

Before evaluation of PSG, all patients completed the 11-item self-report BQ (divided into 3 categories), which aimed to detect important symptoms for the diagnosis of OSA. Subjects placed in at least in two positive categories were considered at high risk for OSA. In category 1, positive score was defined as persistent symptoms (3 to 4 times/wk) in two or more questions about their snoring. In category 2, positive score was defined as persistent (3 to 4 times/wk) wake time sleepiness, drowsy driving, or both. In category 3, positive score was defined as a history of high blood pressure or a BMI ≥ 30 kg/m^2^ [13]. Those who denied having symptoms or who qualified for only one symptom category were placed in the lower risk group [13, 14, 23].

#### Epworth sleepiness scale

Epworth Sleepiness Scale (ESS) is a questionnaire to detect daytime sleepiness, consisting of a simple subjective scale and covering 8 different daytime situations. Subjects were asked to rate on a scale of 0 (would never doze) to 3 (high chance of dozing) how likely they would be to doze off or fall asleep in the 8 situations, based on their recent everyday life. A distinction was made between dozing off and simply feeling tired. Thus, the scale ranged from 0 to 24, and excessive daytime sleepiness was defined when the score is ≥11 [19, 24].

### Statistical analysis

**Fig 1:**
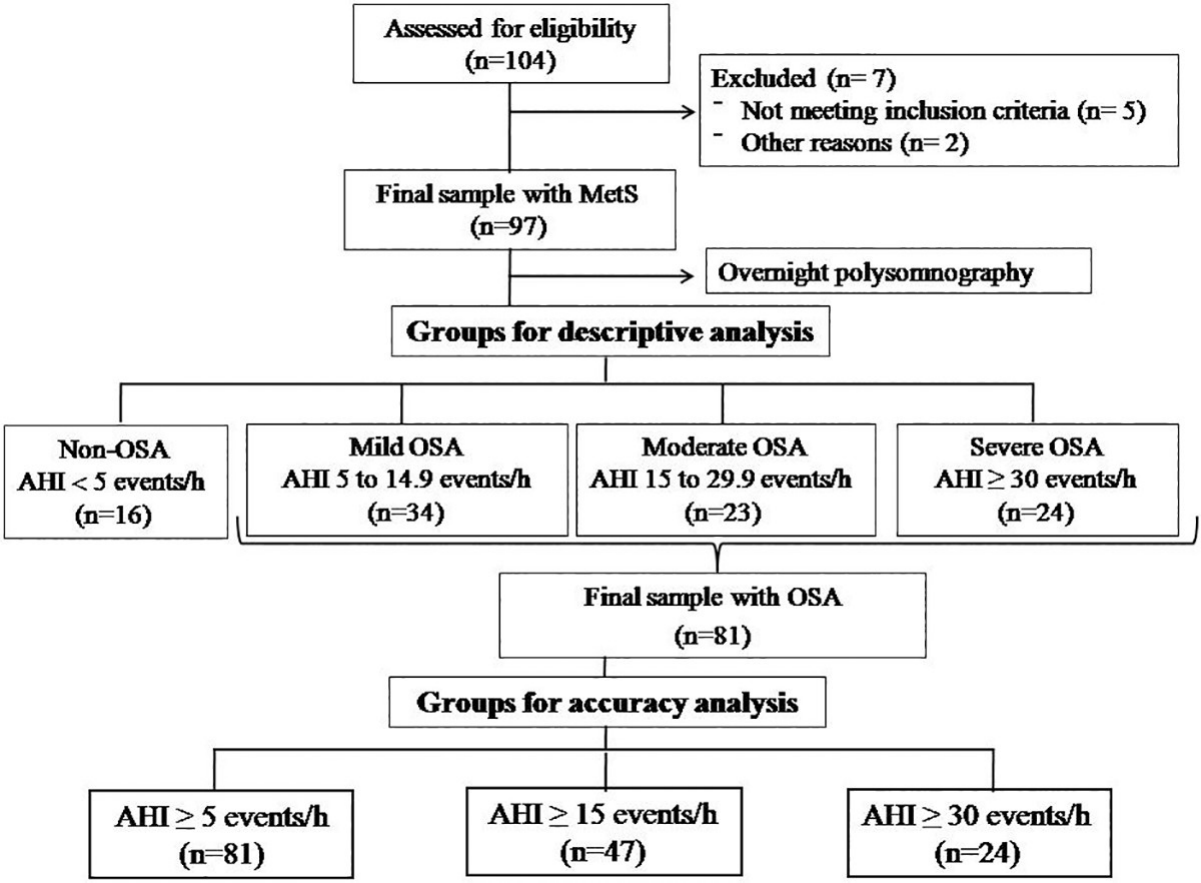
Participants flow chart and study overview. Note that there are 2 different cutoffs of groups based on apnea/hypopnea index (AHI) according to Obstructive Sleep Apnea (OSA) severity: one for descriptive analysis of all Metabolic Syndrome (MetS) patients and other for those MetS with OSA for accuracy analyses of the Berlin Questionnaire as a screening tool when compared to the Polysomnography.

The data are presented as a mean±standard error (SE). For the analysis, MetS patients were divided in two different ways according to OSA severity, based on the AHI cutoffs obtained from PSG (**Fig 1**).

First, for the descriptive analysis of data we divided MetS patients into 4 groups: severe OSA (AHI ≥ 30 events/h), moderate OSA (AHI from 15 to 29.9 events/h), mild OSA (AHI from 5 to 14.9 events/h) and non-OSA (AHI < 5 events/h). For MetS risk factors (waist circumference, glucose, triglycerides, HDL-c, systolic and diastolic blood pressure) and PSG measurements (AHI, arousal index and minimum SaO_2_) we performed the Kolmogorov–Smirnov and Levine tests to assess the normality and homogeneity for each variable studied. We used one-way analysis of variance (ANOVA) followed by Scheffé *post hoc* test for multiple comparisons among the four groups.

Second, to determine accuracy, sensitivity, specificity, positive and negative likelihood ratios and positive and negative predictive values of the BQ screening tool when compared to the PSG (S1 Table), we divided the MetS+OSA into 3 groups for the following AHI cutoffs: AHI ≥ 5 events/h, including all MetS patients presenting OSA at any level, i.e., mild, moderate and severe OSA; AHI ≥ 15 events/h, including all patients with moderate and severe OSA; and AHI ≥ 30 events/h, including only patients with severe OSA. For data analysis we used the Altman’s Kappa Benchmark Scale (< 0.20 was considered poor, 0.21 to 0.40 as fair, 0.41 to 0.60 as moderate, 0.61 to 0.80 as good, 0.81 to 1.00 as very good) to codify in a logical way as a concrete representation of the abstract concept of validity coefficient of the results [25]. To determine the practical significance of the screening measures, receiver operating characteristic (ROC) curve analysis was performed to evaluate the area under the curve (AUC), comparing clinical variables to BQ for prediction of OSA. For all comparisons, significance level was set at P<0.05.

## Results

We initially recruited 104 patients, but seven patients were excluded, since five of them did not meet the inclusion criteria and two got pregnant. According to OSA severity, our final sample (n = 97) was first classified as follows: 24 (24.7%) were found to be severe, 23 (23.7%) moderate, 34 (35.1%) mild and 16 (16.5%) non-OSA, as shown in **Fig 1**.

Baseline characteristics, MetS risk factors and polysomnography measurements of the groups based on AHI cutoffs for OSA severity are shown in **Table 1.** Regarding gender distribution, there were more males, as expected, in severe and moderate OSA groups than in mild and non-OSA groups. There were no differences among groups in age, BMI, glucose, triglycerides, HDL-c, systolic and diastolic blood pressure. However, severe OSA patients had higher waist circumference than mild OSA.

**Table 1:** Baseline characteristics, metabolic syndrome risk factors and polysomnography measurements (absolute and prevalence values) in groups based on AHI cutoff for OSA severity groups: severe (AHI ≥ 30 events/h), moderate (AHI from 15 to 29.9 events/h), mild (AHI from 5 to 14.9 events/h) and non-OSA (AHI < 5 events/h).

	OSA			
	Severe (n = 24)	Moderate (n = 23)	Mild (n = 34)	Non- (n = 16)
**Characteristics**				
Age, y	47±2	49±2	47±1	46±1
Sex, M/F	17/7^†^^‡^	15/8^†^^‡^	14/20	2/14
BMI, kg/m^2^	33±1	32±1	32±1	32±1
**Metabolic Syndrome Risk Factors and Prevalence Values**				
WC, cm	109±2^‡^	107±2	102±1	104±2
	92%	96%	79%	100%
Glucose, mg/dL	103±2	106±2	98±1	97±3
	67%	78%	38%	44%
Triglycerides, mg/dL	201±16	172±19	168±15	180±30
	75%	57%	56%	50%
HDL-c, mg/dL	41±2	40±2	44±2	41±2
	71%	78%	68%	94%
SBP, mmHg	132±3	128±3	127±2	131±5
	88%	70%	65%	69%
DBP, mmHg	87±2	86±2	86±2	86±3
	88%	70%	65%	69%
**Polysomnography Measurements**				
AHI, events/h	61±5^†^^,^^‡^^,^^§^	21±1^†^^,^^‡^	9±1	2±1
Arousal index, events/h	36±5^†^^,^^‡^^,^^§^	19±2	14±2	11±2
Minimum SaO_2_, %	77±2^†^^,^^‡^^,^^§^	82±1^†^	86±1	91±1
Epworth Sleepiness Scale, score	10	9	10	8

Values are mean ± standard error. OSA, obstructive sleep apnea group; BMI, body mass index; WC, waist circumference; HDL-c, high-density lipoprotein cholesterol; SBP, systolic blood pressure; DBP, diastolic blood pressure; AHI, apnea-hypopnea index; SaO_2_, oxygen saturation.

† P <0.05 versus non-OSA

‡*P*<0.05 versus mild; and

§*P*<0.05 versus moderate.

Besides higher AHI, the severe OSA group had higher arousal index and lower minimum oxygen saturation than moderate, mild and non-OSA. The arousal indexes were similar among moderate, mild OSA and non-OSA groups. Moreover, moderate OSA subjects presented lower levels of minimum oxygen saturation than those non-OSA. No differences were found between mild and non-OSA in PSG studied variables. Similarly, there were no differences among groups regarding ESS **(Table 1)**.

The performance of the BQ for OSA in patients with MetS is shown in **Table 2**. Of the 97 patients with MetS included in this study, the BQ identified risk for obstructive sleep apnea in 63 patients (65%).

**Table 2:** Performance of the Berlin Questionnaire to predict OSA in patients with MetS in groups based on AHI cutoff for OSA severity groups: all MetS patients with OSA (AHI ≥ 5), in MetS patients with moderate-severe OSA (AHI ≥ 15 events/h), and in MetS patients with severe OSA (AHI ≥ 30 events/h).

OSA (events/h)	Sensitivity (95% CI)	Specificity (95% CI)	PPV (95% CI)	NPV (95% CI)	LR+	LR-	Accuracy % (95% CI)	ROC % (95% CI)
AHI ≥ 5	0.65 (0.54–0.76)	0.38 (0.15–0.65)	0.84 (0.73–0.92)	0.18 (0.7–0.35)	1.05	0.92	84 (75–90)	67 (0.55–0.79)
AHI ≥ 15	0.73 (0.58–0.85)	0.40 (0.27–0.55)	0.56 (0.39–0.64)	0.64 (0.45–0.79)	1.23	0.66	48 (38–59)	73 (0.58–0.89)
AHI ≥ 30	0.91 (0.72–0.99)	0.42 (0.31–0.54)	0.33 (0.22–0.46)	0.94 (0.80–0.99)	1.25	0.21	25 (17–25)	65 (0.33–0.97)

AHI, apnea-hypopnea index; PPV, positive predictive value; NPV, negative predictive value; LR+, positive likelihood ratio; LR -, negative likelihood ratio; ROC = receiver operating characteristic.

For all MetS patients with OSA (AHI ≥ 5 events/h, n = 81), the BQ showed good sensitivity (0.65), fair specificity (0.38), very good positive predictive value (0.84) and poor negative predictive value (0.18). Similarly, for moderate-severe OSA (AHI ≥ 15 events/h, n = 47) we found good sensitivity (0.73) and fair specificity (0.40). Interestingly, for severe OSA (AHI ≥ 30 events/h, n = 24) we found high sensitivity (0.91) and moderate specificity (0.42) **(Table 2)**. In addition, in patients with MetS, the accuracy of the BQ for predicting OSA was very good (84%) for AHI ≥ 5 events/h, moderate (48%) for AHI ≥ 15 events/h, and fair (25%) for AHI ≥ 30 events/h **(Table 2)**.

**Fig 2:**
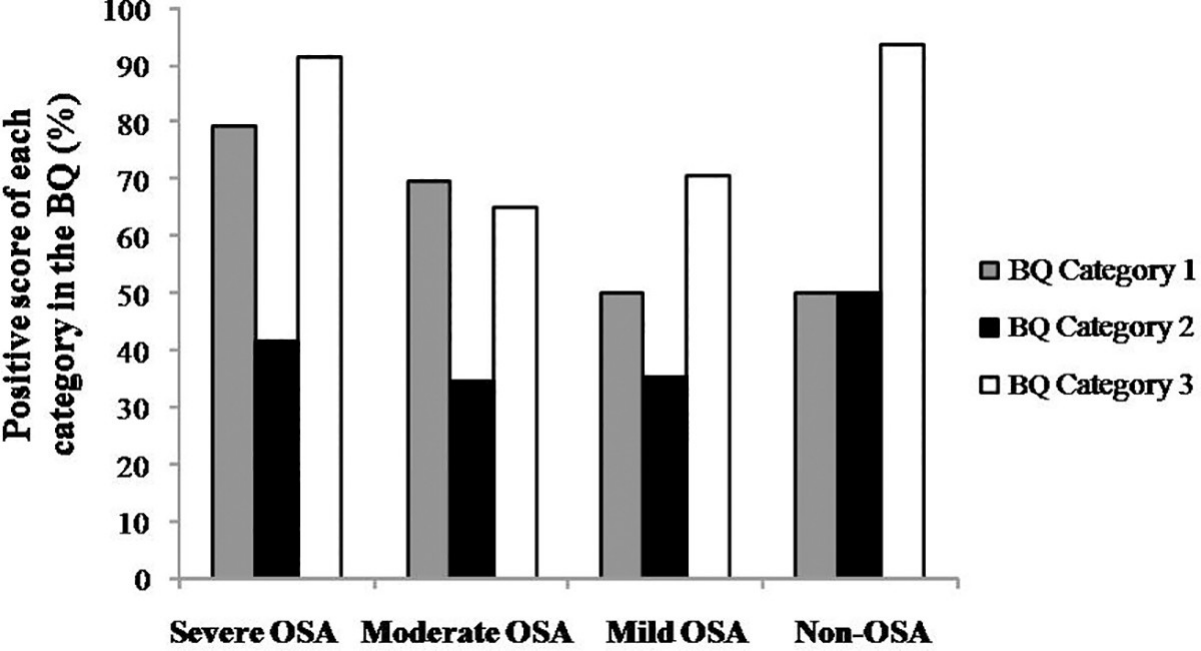
Percentage of patients with positive score for each category in Berlin Questionnaire(BQ) for groups of Metabolic Syndrome patients according to Obstructive Sleep Apnea (OSA) severity: severe (apnea/hypopnea index, AHI ≥ 30 events/h), moderate (AHI from 15 to 29.9 events/h), mild (from 5 to 14.9 events/h) and non-OSA (AHI < 5 events/h). Category 1 is related to positive snoring score if ≥ 2 points; Category 2 is related to positive daytime fatigue and sleepiness if ≥ 2 points; and Category 3 is related to score 1 point for ‘Yes’ in the presence of high blood pressure or if the BMI is greater than 30 kg/m^2^.

**Fig 2** shows the percentage of positive score for each category in BQ for all MetS patients according to OSA severity groups: severe, moderate, mild and non-OSA. Note that in the Category 1, related to snoring, we found positive score in79% of severe OSA, 70% of moderate, 50% of mild and 50% of non-OSA. For Category 2, related to daytime fatigue and sleepiness, we found positive score in 42% of severe OSA, 35% of moderate, 35% of mild and 50% of non-OSA. For Category 3, related to high blood pressure and/or BMI ≥ 30 kg/m^2^, we found positive score in 92% of severe OSA, 65% of moderate, 71% of mild and 94% of non-OSA **(Fig 2)**.

**Fig 3:**
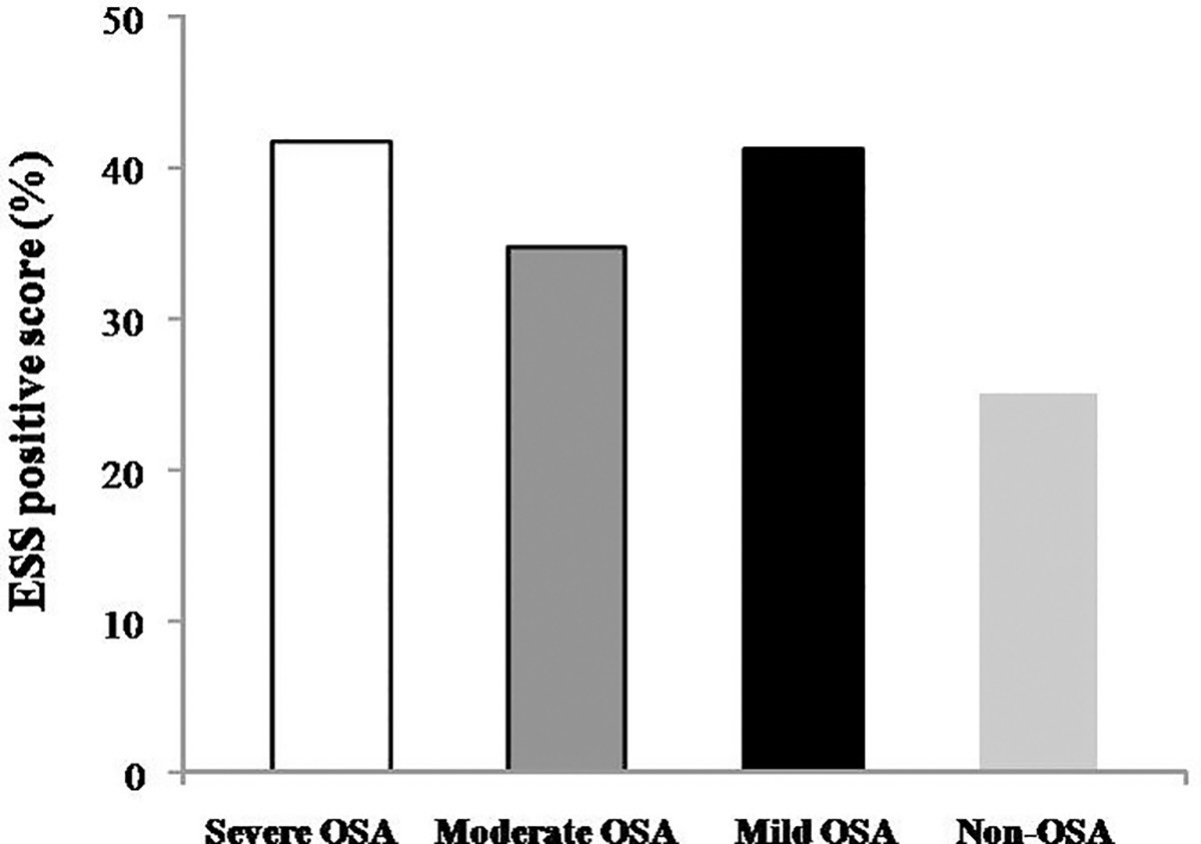
Percentage of patients with positive score (score ≥ 11 points from a scale ranging from 0 to 24) in Epworth Sleepiness Scale (ESS). ESS rates from 0 (would never doze) to 3 (high chance of dozing) in 8 life situations. Studied groups according to Obstructive Sleep Apnea (OSA) severity in MetS patients were divided in severe (AHI ≥ 30 events/h), moderate (from 15 to 29.9 events/h), mild (AHI from 5 to 14.9 events/h) and non-OSA (AHI < 5 events/h).

**Fig 3** presents the percentage of ESS positive score (≥ 11) according to OSA severity cutoff in MetS groups: severe, moderate, mild and non-OSA. The severe OSA group had 42%, moderate 35%, mild 41% while those non-OSA obtained 25% of ESS ≥ 11 points.

## Discussion

The primary finding of this study was that BQ is valid tool for screening OSA in MetS patients. Particularly in patients with severe OSA, BQ has been shown to have high sensitivity for OSA screening (91%). The important clinical implication of this finding lies in the fact that it reinforces the validity of screening for OSA with BQ in patients with MetS, since AHI ≥15 and ≥30 events/h are usually considered clinically relevant and indicate that treatment is needed [26].

PSG is required for confirmation of OSA, even in the presence of home sleep testing using type 3 (portable testing limited to sleep apnea) or other devices. In the present study, we found that BQ in general has good sensitivity (0.65) and fair specificity (0.38) when the cutoff was AIH ≥ 5 events/h, suggesting that it is a valid way of screening OSA in MetS patients.

A recent systematic review and meta-analysis [26] surveyed 35 eligible articles and demonstrated that the usefulness of BQ diagnosis depended on the population studied, since age, gender differences, BMI, and the presence of comorbidities may affect the accuracy of this screening [12]. In addition, sensitivity and specificity varied across studies as they adopted different hypopnea criteria and different thresholds of the apnea-hypopnea index. In line with our findings, the authors found that, in general, BQ had good sensitivity and it was higher at higher AHI thresholds [26].

In fact, several other studies have demonstrated the usefulness of the BQ in different groups of subjects, such as primary care [13], asthmatic [27] and hypertensive patients [28].

In primary care patients, Netzer et al. [13] found that the BQ is a valid tool for identifying patients with OSA. Using an OSA cutoff of AHI ≥ 5, they found that the sensitivity was 86% and specificity was 77% [13]. Similarly, our findings point that both sensitivity and specificity seem to increase from any OSA to severe OSA. Although sensitivity and specificity are usually inversely proportional, specificity and sensitivity of a quantitative test depend on a cutoff value. In a situation when the cutoff is reduced, most people with the disease would be correctly identified, but at the same time the number of false positives would be increased. On the other hand, raising the cut-off value would show more false negatives, but would reduce the number of false positives. However, minimizing false positives and false negatives at the same time maximizes sensitivity and specificity. For a test to be accurate, both sensitivity and specificity should be high. When measuring sensitivity, we only calculate those people with confirmed diagnoses of the disease. High sensitive test detects a high percentage of positive cases, while missing few [29].

A study carried out by Karakoc et al. [18] have investigated the usefulness of the BQ for screening at-risk patients for OSA using a cut-off point of AHI ≥ 5 and found higher sensitivity than ours (83.4% vs. 65%), but lower specificity (22.2% vs. 38%), lower positive predictive value (76.4% vs. 84%) and higher negative predictive value (30.8% vs. 18%) [18]. In the same study, the similarity with our findings is more evident in the higher levels of OSA. For AHI ≥ 15 events/h cutoff, the researchers found that sensitivity was 89.3% and the specificity was 22.6%, while the values in our study ranged from 73% and 91% for sensitivity and 40% and 42% for specificity) [18]. Nevertheless, our data are in line with a large study involving 1,450 in patients in a sleep clinic^,^ [16], in which BQ showed high sensitivity (80% vs. 91% in our study) when the cutoff was IAH ≥ 30 events/h [16].

However, some previous studies have not found BQ useful to identify individuals with OSA. For example, in a study involving patients with recent myocardial infarction, the authors concluded that BQ is not valid in identifying OSA in these patients. Although they found similar sensitivity (0.68) and specificity (0.46) for AHI ≥ 5, the sensitivity did not change from lower to higher AHI (0.68, 0.65 and 0.71), while ours increased (0.65, 0.73 and 0.91). Moreover, in this study of patients with recent myocardial infarction, the positive predictive value (for AHI≥5) was 0.50 vs. 0.84 and the accuracy was 63% vs. 84% in the present study [14].

The negative health outcomes of undiagnosed severe OSA patients add to the importance of early screening for this condition. Thus, the main message and the clinical importance of the present study lie in the fact that a simple tool such as BQ may help to decide which patients must undergo PSG. This is particularly true for severe OSA (IAH ≥30 events/h), with BQ presenting higher sensitivity (91%), moderate specificity (42%) and higher negative predictive value (94%).

The validity of BQ as a screening tool for OSA in MetS was reinforced by the very high association between both conditions, MetS and OSA. Indeed, we found that 84% of MetS patients also had OSA (IAH ≥ 5 events/h). Our data corroborate previous studies that found, in general, a high prevalence of OSA in MetS patients [3–5]. However, reported prevalence may vary, as it depends upon the cutoff used and the percentage of the oxygen desaturation used (3% vs 4%) to determine hypopnea. However, studies with similar criteria of ours, i.e., considering moderate to severe OSA (AHI≥ 15 events/h) and oxygen desaturation of 3%, have reported higher prevalence of OSA in patients with MetS. For instance, Drager et al. [30] have reported that the prevalence of moderate and severe OSA was 68% in MetS patients [30]. In a similar population and with similar criteria, Ambrosetti et al. [17] have found 53% of OSA [17] In the present study we have found 48% of moderate and severe OSA, still a remarkable high prevalence of OSA in MetS patients. Considering the high prevalence of OSA in MetS, all patients should undergo to a complete PSG. However, feasibility and costs are important factors to be considered in the clinical decision. Therefore, the physician's judgment for the indication of PSG should take into account all possible clinical evidence pointing to the risk of OSA. An alternative to reduce the cost of disposable accessories required and the costs of time spent by medical staff is the PSG at home using portable monitoring equipment, which is a reliable tool for diagnosing patients referred for evaluation of OSA [31]. Since the overlap between MetS and OSA potentiates pathophysiologic alterations, leading to higher BP, higher sympathetic drive, diminished baroreflex sensitivity [4], and increased chemoreflex sensitivity [21], early diagnosis of the OSA is critical to determine the optimal clinical management to prevent cardiovascular diseases.

It is also important to focus on each category of BQ. The severe OSA had higher positive score in Category 1, followed by moderate, mild and non-OSA. This category covers snoring intensity and frequency. Similarly, Maimon et al. [32] have observed a significant positive correlation between the severity of the OSA and snoring intensity [32]. Furthermore, Drager et al. [28] have observed a high score in snoring category of BQ in patients with hypertension and OSA [28]. These findings show that snoring is an important OSA-associated symptom.

In line with other investigations, we did not find any differences among groups regarding reported excessive daytime sleepiness either in ESS or in BQ Category 2. In our study, sleepiness was the category the least likely to be the determining factor for risk in the classification OSA among MetS patients. Thus, these responses should be interpreted with caution, and the excessive daytime sleepiness reported in the ESS and in Categories 2 of BQ might not represent an index in the OSA screening of MetS patients. Further studies should be undertaken to determine the clinical significance of the excessive daytime sleepiness in MetS patients.

In our study population, as expected, and despite OSA severity, we found high score levels in Category 3 of the BQ. In fact, both obesity and hypertension are risk factors for MetS and they are viewed as the major risk factors associated with OSA. Obese individuals often have increased deposition of fat on the neck [33]. This alteration may cause the airway to collapse during sleep, leading to the classical symptoms of OSA, such as snoring, apneas and hypopneas. Regarding hypertension, there is strong evidence in the literature linking OSA to both essential and resistant hypertension [34, 35]. Furthermore, in the prospective Wisconsin Sleep Cohort Study, the authors found a dose–response association between previous sleep-disordered breathing and the presence of hypertension after four year of follow-up, regardless of known confounding factors [36]. Indeed, we have shown in a previous study [4] that in recent diagnosed MetS patients, the comorbid OSA led to higher BP, higher sympathetic drive, and diminished baroreflex sensitivity, when compared to patients with MetS non-OSA [4].

Therefore, in the present study the high prevalence in BMI and BP (category 3) and the presence of snoring symptoms (category 1) suggest that categories should be considered the best clinical parameter in the screening of OSA in MetS patients.

The mechanisms underlying the association between OSA and MetS have yet to be fully understood. However, some characteristics are frequently present in OSA. For instance, OSA is more common in men [6]. In our study population, severe and moderate OSA groups have more males than in mild or non-OSA. As expected, the group non-OSA had more women. In addition, although not-statistically significant, the levels of fasting glucose exceeded the cutoff of the normality level (100mg/dL) in MetS with severe and moderate OSA.

Therefore, the present study has provided convincing evidence that snoring symptoms and presence of increased BMI and BP (Categories 1 and 3, respectively) should be considered user-friendly diagnostic tools in the screening of OSA in MetS patients. Therefore, based on our findings and on the evidence from literature, we may recommend BQ as an appropriate tool for screening the risk of OSA in MetS patients.

Our study suggests that in patients with MetS, the need to refer to PSG in diagnosing OSA may be addressed by the results of the BQ, a simple and easy screening questionnaire for OSA.

Finally, we would like to emphasize the importance of early diagnosis and treatment of OSA, since obstructive sleep apnea is highly prevalent in MetS patients. The subjectivity of the symptoms should not be neglected, and the BQ is a feasible, useful and valid tool to help in this task.

Our study has some strengths and limitations that need to be addressed. The main strengths lie in the use of full PSG to diagnose OSA and the careful selection of MetS patients who were not receiving any medication. In addition, unlike other studies which recruited participants from sleep laboratories and therefore with higher chance of having OSA, the patients in the present study were selected exclusively from an outpatient clinic, without prior knowledge of whether or not they had OSA. We should also point several limitations in the present study. First, the use of a convenience sample is a limitation of our study. Second, we found significant difference in gender distribution among different OSA severity groups, with more males in severe and moderate OSA groups. However, this distribution reflects the reality that sleep-disordered breathing is more prevalent in men than in women [37]. Third, the present result may not be extrapolated to patients with MetS undergoing drug-based treatments, with severe hypertension or morbid obesity. Finally, additional studies with larger samples and the inclusion of other relevant questionnaires would be important to assess the better tool for screening the risk of OSA in patients with MetS.

## Supporting information

S1 Table Data to determine accuracy, sensitivity, specificity, positive and negative likelihood ratios and positive and negative predictive values of the Berlin Questionnaire (BQ) as a diagnostic screening for obstructive sleep apnea (OSA) in metabolic syndrome (MteS) patients. (DOCX)
