# Supplementary material for: Identifying the risk of obstructive sleep apnea in metabolic syndrome patients: Diagnostic accuracy of the Berlin Questionnaire
Source: PLoS One. 2019 May 21;14(5):e0217058. doi: 10.1371/journal.pone.0217058 (PMC6528986; doi:10.1371/journal.pone.0217058)
Supplement: S1 Table — (DOCX) [file pone.0217058.s001.docx]

**Data Availability Statement:** All relevant data are within the manuscript and its Supporting Information files.

**Supporting information**

**S1 Table. Data to determine accuracy, sensitivity, specificity, positive and negative likelihood ratios and positive and negative predictive values of the Berlin Questionnaire (BQ) as a diagnostic screening for obstructive sleep apnea (OSA) in metabolic syndrome (MetS) patients.**

|  | **AHI ≥ 5** | **AHI ≥ 15** | **AHI ≥ 30** |
| --- | --- | --- | --- |
| **True positive** | 53 | 33 | 21 |
| **False positive** | 10 | 31 | 43 |
| **True negative** | 6 | 21 | 31 |
| **False negative** | 28 | 12 | 2 |
| **Total** | 97 | 97 | 97 |
